# An Item Response Theory-Based Validation of the Vanderbilt Classroom Listening Assessment Short Survey for Children With Unilateral Hearing Loss

**DOI:** 10.1097/AUD.0000000000001897

**Published:** 2026-10-14

**Authors:** Amy Liang, Haiping Huang, Sonya K. Sterba, Erin M. Picou

**Affiliations:** 1 Quantitative Methods and Data Analytics, Vanderbilt University, Nashville, Tennessee, USA; 2 Department of Hearing and Speech Sciences, Vanderbilt University Medical Center, Nashville, Tennessee, USA.

**Keywords:** Children, Hearing loss, Item response theory, Listening difficulties, Psychometric, Validation

## Abstract

**Objectives:**

The Vanderbilt Classroom Listening Assessment Short Survey (VCLASS) was previously developed as a tool to monitor listening difficulties experienced by school-aged children in classrooms. However, the previous statistical approach used in the initial validation did not fully evaluate the questionnaire’s psychometric properties, did not take into account the ordinal nature of responses, and did not contrast hearing-aided versus hearing-unaided conditions. The present study aimed to evaluate the psychometric properties of the 15‑item VCLASS using item response theory (IRT) in both hearing-aided and unaided conditions. More specifically, we aimed to (1) assess the latent structure for each listening condition (unaided and aided); (2) determine appropriate IRT-based models to estimate item discrimination and threshold parameters for each listening condition; (3) assess reliability from the IRT-based model; and (4) evaluate both uniform and nonuniform age-related differential item functioning (DIF) under each listening condition.

**Design:**

We applied IRT-based analyses to analyze responses previously collected from children aged 9 to 17 years with unilateral hearing loss. All children (n = 1148) completed the VCLASS imagining they were not using a device (unaided); most children (n = 897) also completed the VCLASS imagining they were using their personal listening device (aided). For each listening condition (unaided, aided), dimensionality of VCLASS items was assessed taking into account the ordinal nature of responses. Next, the best-fitting models were used to estimate item discrimination (*â*) and threshold parameters (*β̂*_1_ to *β̂*_4_), to evaluate item and person fit, and to examine reliability. Last, age-related uniform and nonuniform DIF were assessed for each listening condition.

**Results:**

Parallel analysis based on polychoric correlations supported a two-factor structure for both the hearing-unaided and hearing-aided conditions. In both conditions, response patterns were largely captured by a dominant, general school-related listening-difficulty factor; additionally, one item (when unaided) or four (when aided) items loaded on a specific/minor factor representing school-related close-proximity listening difficulty. The two-factor graded response model provided better model fit relative to the two-factor generalized partial credit model. All items showed good fit and high discrimination. IRT-based marginal reliability for factor score estimates was strong (unaided: 0.88 and 0.89; aided: 0.86 and 0.85 for factors 1 and 2, respectively). Test information curves indicated greatest measurement precision where there were the most sample respondents—which included low, middle, and high, but not extreme, levels of listening difficulty—with comparable profiles across listening conditions. Age-related DIF effects on item responses were negligible, supporting age-invariant measurement.

**Conclusions:**

The IRT-based validation confirms that the VCLASS provides a reliable and consistent measure of classroom listening difficulty across unaided and aided listening conditions in children with unilateral hearing loss. By considering the ordinal nature of the data in both modeling and missing data imputation, current results support a two-factor structure across unaided and aided conditions. We also demonstrated that the VCLASS has strong item functioning and trivial age‑related bias. The VCLASS provides listening difficulty scores on an interval‑level continuum for classroom listening difficulties, supporting its use in clinical and research settings for children with unilateral hearing loss.

## INTRODUCTION

Unilateral hearing loss affects 3 to 14% of school-aged children (Ross et al. 2010; Lieu 2018). These individuals experience hearing loss in one ear and thus may be negatively impacted in their abilities to localize sound and understand speech in background noise (Reeder et al. 2015). UHL in children is also associated with increased listening-related effort and fatigue compared to peers with normal hearing (Bess et al. 2020; Oosthuizen et al. 2021a; Carpenter et al. 2022). Both skills of sound localization and speech understanding in noise are essential to successfully navigate busy classroom settings (Bovo et al. 1988; English & Church 1999).

It is perhaps not surprising that children with UHL are at risk for language delay and educational problems (Bess & Tharpe 1986; Lieu 2013; Davis et al. 2024). Therefore, there is a critical need for tools that could accurately and reliably measure school-based hearing challenges among children with UHL. Moreover, the desired tool should be able to capture listening difficulties in the classroom both with and without devices. Current UHL interventions may have differential benefits at different talker-of-interest locations. For example, contralateral routing of signal systems and bone conduction hearing aids may be most beneficial in overhearing situations (Liu et al. 2017; Oosthuizen et al. 2021b), while cochlear implants can provide better localization cues for a wider range of talker locations (Buss et al. 2018). Given the varied benefits across types of interventions and their apparent difference from unaided listening, it is essential to evaluate the psychometric performance of the tools in both unaided and aided conditions.

Standard hearing assessments, like those in laboratory settings, fall short of fully simulating real-world acoustic complexity by not including these classroom-specific factors (Bess & Tharpe 1986; Kenworthy et al. 1990). Subjective questionnaires play an important role in the identification and management of UHL (Bagatto et al. 2019). Although many existing self-report measures such as the Children’s Home Inventory for Listening Difficulties (Anderson & Smaldino 2000) and the HEAR-QL Quality of Life Questionnaire (Umansky et al. 2011) capture general listening difficulties, they are primarily designed for home or social contexts and overlook critical classroom experiences. Some examples of these classroom-specific factors that students might face include listening to talkers at a distance, dynamic speaker locations, and multiple competing noise sources (Purcell et al. 2018).

To address the need for a measure specifically focused on school-based scenarios, Picou et al. (2020) developed a pilot version of *the Vanderbilt Classroom Listening Assessment Short Survey (VCLASS*), which included 15 items that describe real-world, school-based listening scenarios. In a pilot study of 20 children aged 10 to 16 years, the instrument captured substantial individual variation in self-reported listening difficulty. Using a revised version of the VCLASS and responses from n = 1148 children aged 9 to 17 with UHL, exploratory factor analysis and classical reliability indices indicated excellent internal consistency and reliability (Picou et al. 2025).

Focusing only on the unaided data, Picou and colleagues used a classical test theory (CTT) approach to validate the VCLASS and relied on Pearson’s correlations (assuming continuously distributed items) in both data analyses and in multiple imputation procedures, despite items being ordinal. CTT indices alone cannot examine sample-invariant item parameters or measurement precision across the full continuum of classroom hearing challenges. By contrast, item response theory (IRT) provides a framework to model how individual differences in a latent trait relate to item responses and provides information functions that capture measurement precision across different levels of a latent trait (e.g., Embretson & Reise 2000; de Ayala 2009).

Benefits of an IRT approach have been demonstrated using different questionnaires. For example, IRT analyses of the Patient Health Questionnaire-9 identified variability in item discrimination and improved understanding of where the scale provides the most information, beyond what CTT reliability indices suggested (Forkmann, Boecker, Norra, et al. 2009; Forkmann, Boecker, Wirtz, et al. 2009). The Short Form Health Survey-36 suggested multidimensionality and improved scoring precision when evaluated with IRT compared to traditional reliability indices (Ware et al. 1999; Reise & Revicki 2015; Lins & Carvalho 2016). Likewise, in the Alcohol Use Disorders Identification Test, CTT suggested unidimensionality and acceptable reliability, but IRT analysis highlighted that items functioned differently at lower versus higher severity levels of alcohol problems, which helped refine cutoffs for screening (Paulus et al. 2022). These examples show how IRT can refine and extend conclusions from CTT and thus might be beneficial for further evaluation of the VCLASS.

In the present study, we extended Picou and colleagues’ work by conducting an IRT validation of the 15-item VCLASS scale using a full dataset of 2045 responses from 1148 school-age children (aged 9 to 17 years) with UHL. These responses included aided data (n = 897), whose psychometric properties were not previously examined, and unaided data (n = 1148), whose psychometric properties were previously evaluated only using CTT. Unique contributions of this study also include appropriately accommodating the ordinal nature of the item responses in both modeling and in multiple imputation procedures. Within our IRT framework, classroom listening difficulty is conceptualized as a latent trait, which captures a child’s self-reported ability to listen and understand auditory information in school-based listening situations.

## MATERIALS AND METHODS

### Participants and Data

Data were collected for the previously reported evaluation (Picou et al. 2025). In brief, participants were recruited via email. Following informed consent of their parents, child participants with reported UHL provided informed assent and then completed the VCLASS, first as if they were not wearing any hearing devices (unaided) and then as if they were wearing their hearing devices, if applicable (aided). Children were school-aged (with mean = 13.35 years, median = 13, 1st quantile = 12, 3rd quartile = 15, IQR = 3, and SD = 1.94 years). The data collection phase was completed in 9 days between March 11 and March 19, 2021. The final dataset included 2045 survey responses from children aged 9 to 17 years with UHL. Among these children, the parent-reported degree of hearing loss was: A little loss: 136 (11.8%); Some loss: 446 (38.9%); Significant loss: 404 (35.2%); Total loss: 162 (14.1%). Within these responses, 1148 completed the unaided version of the VCLASS, and 897 completed the aided version while using personal hearing devices. Only the former unaided responses were included in the previous CTT psychometric analysis (Picou et al. 2025).

### The VCLASS Instrument

The VCLASS (available in full here: https://osf.io/j793s/) includes 15 items, each describing a scenario for classroom listening conditions (e.g., hearing a teacher from the back of the room, overhearing classmates, localizing someone calling one’s name in a noisy gym). For each item, there is a short description and picture that shows the speaker and listener locations. Children can select how “easy” or “hard” each scenario is, on a 5-point Likert scale, with an option of “Does Not Apply” for non-relevant situations. In the original CTT-based validation of the only-unaided responses, three subscales were identified: distant listening (Far; items 3, 7, 8, 12, 14, and 15), close-proximity listening indoors (Near Inside; items 1, 2, 4, and 9), and close-proximity listening outdoors (Near Outside; items 5, 10, and 13; Picou et al. 2025). Items 6 and 11 contributed only to the total score. Subscale and total scores were calculated by averaging applicable items. Because the original analyses treated the five-category responses as continuous (rather than ordinal) during analysis and missing data imputation, our present study re-evaluated the latent structure using ordinal-data methods and polytomous IRT models in both unaided and aided conditions during analyses as well as missing data handling. Because our latent trait was classroom listening difficulty, we recoded the VCLASS response scale (which originally ranged from 1 = “Very Hard” to 5 = “Very Easy”), to now range from 0 = “Very Easy” to 4 = “Very Hard,” such that higher scores now reflect greater listening difficulty. This reverse-coding does not change our main results or substantive conclusions.* Such a reverse-coding scheme aligns common IRT-based interpretation with our latent construct (where higher item-scores conventionally mean higher levels of the latent trait being measured, i.e., higher listening difficulty in school environments). After reverse-coding, as the latent trait (listening difficulty) increases, the probability of endorsing higher response categories (i.e., choosing “Very Hard” to hear) also increases.

### Analytic Strategy

To further validate the VCLASS instrument, we implemented a four-phase framework for both aided and unaided listening conditions (see Figure 1 in Supplemental Digital Content, https://links.lww.com/EANDH/C40). In phase 1, for each listening condition, we assessed dimensionality using parallel analyses on polychoric correlations (Horn 1965; Glorfeld 1995) and computed supplementary dimensionality indices (Revelle 2025; Revelle & Condon 2025). For descriptive purposes, we also calculated CTT-based item indices for each listening condition. In phase 2, we compared the fit of two candidate polytomous IRT models that measure how well each item differentiates children with different levels of listening difficulties in general and evaluate whether any items show poor psychometric properties. Specifically, the two polytomous IRT models compared were the Generalized Partial Credit Model (GPCM; allows unequal item discriminations) and the Graded Response Model (GRM; allows unequal item discriminations but requires that thresholds be ordered for a given item). In short, these two models each involve *discrimination* parameters and *threshold* parameters but impose different assumptions about these parameters. An *item discrimination* is a measure of each item’s ability to discriminate between various levels of the latent construct; it shows how each item differentiates between children with varying reported listening difficulties. A larger item discrimination indicates the item is sensitive to variations in the latent construct. A *threshold* parameter for an item in the GRM indicates the level of the latent trait needed to have a 50% chance of selecting a given or higher category. A *k*-category item allows estimating *k −* 1 thresholds. We assessed global model fit as well as item- and person-level fit.

After identifying a two-factor GRM as the optimal solution, in phase 3, we used all 15 items to estimate two-factor GRM-based item discrimination and threshold parameters, evaluated using marginal reliability, and generated test information curves to examine measurement precision across the latent trait. The goal of these analyses was to examine the individual item quality and characteristics across all 15 test items and ultimately to identify high-quality items for the VCLASS scale. In phase 4, we probed uniform and nonuniform DIF by age, using ordinal logistic regression in the *mirt* package (Chalmers 2012) and quantifying effect sizes with McFadden’s pseudo-Δ*R*^2^.

## RESULTS

### Phase 1.1: Dimensionality Assessment

For each listening condition (aided, unaided), we first evaluated VCLASS dimensionality using the unidimensionality index (*u*), which shows the ratio of the first eigenvalue to the sum of all eigenvalues (a value of 1.0 indicates unidimensionality). The *u* was 0.90 for the unaided condition and 0.88 for the aided condition (using *psych* R package [Revelle 2025]), indicating that variance was not concentrated exclusively in a single dimension. To further clarify the factor structure, we examined explained common variance (ECV) values, for which ECV > 0.85 indicates unidimensionality (Stucky & Edelen 2014; Rodriguez et al. 2016). The ECV values were 0.77 for the unaided and 0.76 for the aided condition, likewise suggesting that although shared variance was substantial, it was distributed across more than one factor. To further evaluate whether the 15 VCLASS items measure a single underlying construct, for each listening condition, we next used parallel analysis based on polychoric correlations (i.e., treating ordinal data as ordinal) to determine the underlying factor structure (Horn 1965; Glorfeld 1995). As shown in Fig. 1, for each (aided and unaided) listening condition, two factors exceeded the corresponding eigenvalues generated from random data, indicating that the response patterns were better captured by two underlying factors. The dominant factor reflects general classroom listening difficulty, whereas the second factor captures close-proximity listening situations (see Phase 2.2: Parameter estimates from best-fitting IRT model).

**Fig. 1.**
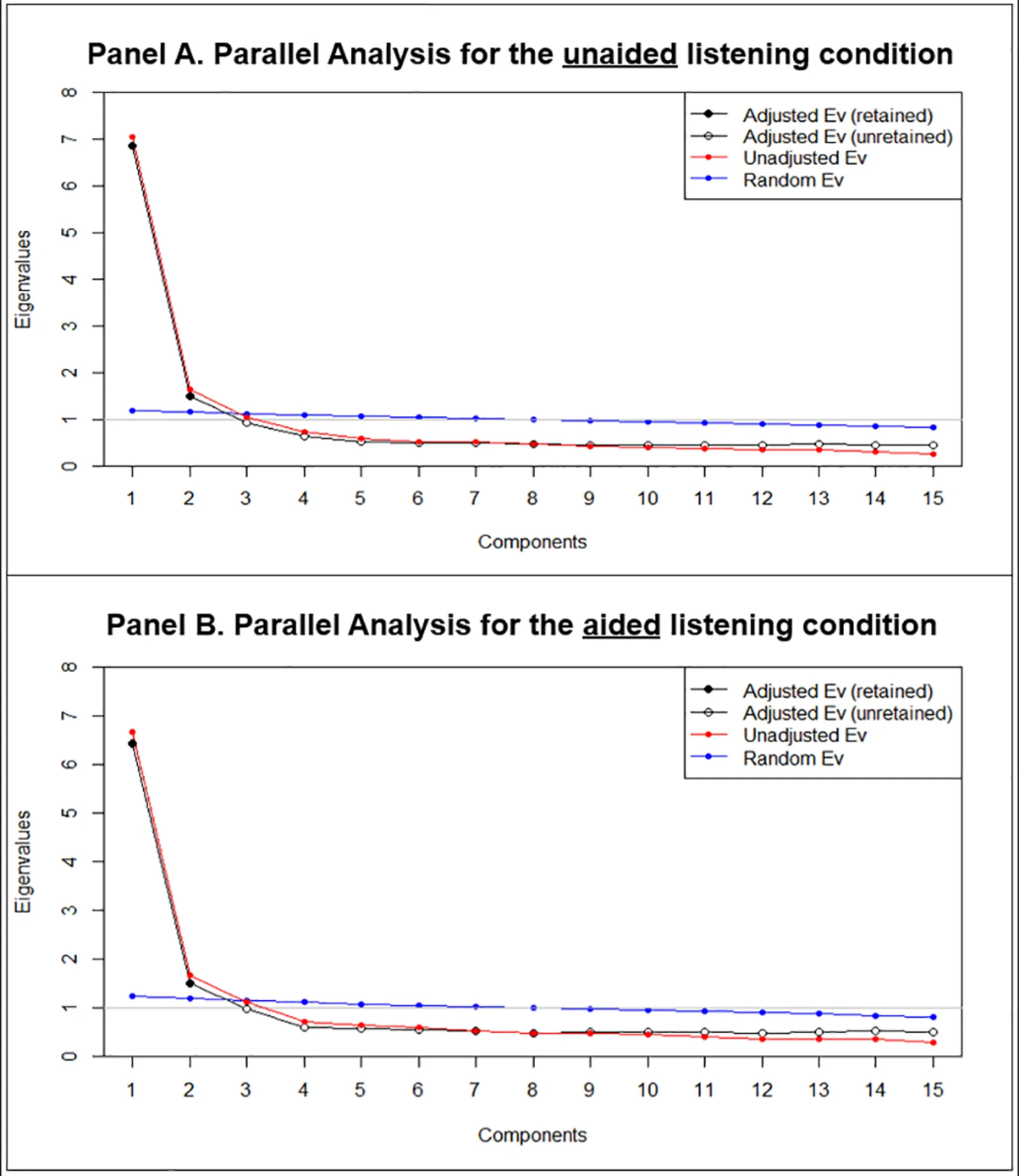
Parallel analysis scree plot for the unaided listening condition (at the top; A) and aided listening condition (at the bottom; B) using polychoric correlations. The horizontal line indicates the eigenvalue of 1. EV indicates eigenvalue.

### Phase 1.2: Preliminary CTT-Based Item Analyses

For descriptive purposes, CTT-based item analyses are presented for the unaided (Table 1) and aided condition (Table 2).

**TABLE 1.** CTT mean response, item-total correlation, and proportions for each response of the VCLASS scale for the unaided dataset

Item	Item Responses (%)						Mean Response	Correlation Between Item Score and Total Test Score
	Very Easy (to Hear)	A Little Easy	Not Easy or Hard	A Little Hard	Very Hard	N/A or Nonresponse		
1	4.007	23.171	16.899	38.763	13.676	3.484	2.337	0.631
2	5.139	27.613	20.209	29.965	14.199	2.875	2.189	0.646
3	2.352	10.889	16.115	33.101	33.188	4.355	2.875	0.536
4	4.617	21.516	24.826	34.669	12.369	2.003	2.242	0.669
5	16.725	37.021	16.638	22.125	5.662	1.829	1.579	0.512
6	2.526	20.209	23.084	35.540	15.592	3.049	2.374	0.597
**7**	1.829	12.369	21.690	35.105	24.477	4.530	2.683	0.657
8	2.091	12.805	23.693	42.334	14.808	4.268	2.539	0.711
9	3.833	27.875	23.606	29.791	11.934	2.962	2.176	0.654
10	2.265	16.028	23.258	39.547	15.767	3.136	2.476	0.658
11	5.052	26.655	20.732	33.885	10.889	2.787	2.133	0.718
12	1.655	7.666	17.073	37.195	32.230	4.181	2.910	0.590
13	3.397	18.206	26.307	33.972	14.111	4.007	2.351	0.561
14	1.916	7.230	13.589	40.854	30.314	6.098	2.932	0.552
15	1.307	12.282	16.115	28.571	33.972	7.750	2.889	0.636

CTT, classical test theory; VCLASS, Vanderbilt Classroom Listening Assessment Short Survey.

**TABLE 2.** CTT mean response, item-total correlation, and proportions for each response of the VCLASS scale for the aided dataset

Item	Item Responses (%)						Mean Response		Correlation Between Item Score and Total Test Score
	Very Easy (to Hear)	A little Easy	Not Easy or Hard	A little Hard	Very Hard	N/A or Nonresponse			
1	48.384	40.245	6.689	2.341	0.669	1.672	0.625	0.551	
2	56.745	34.002	4.571	1.672	1.115	1.895	0.516	0.616	
3	29.431	53.289	11.929	3.902	0.892	0.557	0.911	0.596	
4	44.593	42.921	8.027	2.564	0.557	1.338	0.678	0.663	
5	66.555	25.195	4.348	1.895	0.334	1.672	0.401	0.598	
6	42.475	42.586	10.368	3.456	1.003	0.111	0.755	0.613	
7	29.097	54.181	12.152	3.790	0.446	0.334	0.913	0.659	
8	32.887	52.508	11.037	2.676	0.446	0.446	0.834	0.657	
9	46.042	42.809	6.243	2.676	0.446	1.784	0.651	0.632	
10	30.546	52.731	12.040	2.787	1.226	0.669	0.893	0.672	
11	47.938	40.134	6.689	2.787	0.669	1.784	0.640	0.637	
12	22.854	49.387	20.624	5.909	1.115	0.111	1.120	0.599	
13	38.462	46.042	10.033	3.567	1.226	0.669	0.804	0.616	
14	20.401	57.971	12.932	5.128	3.233	0.334	1.122	0.431	
15	31.550	47.603	11.371	6.020	2.118	1.338	0.969	0.493	

Higher mean score means more children rated the scenario as hard; a higher item-total correlation means the item does a better job of separating children with higher versus lower overall listening-difficulty scores.

CTT, classical test theory; VCLASS, Vanderbilt Classroom Listening Assessment Short Survey.

### CTT-Based Item Analyses on the Unaided Dataset

In the unaided condition, mean response scores ranged from 1.58 to 2.93 on a (recoded) 0 to 4 scale, where a higher score indicates greater perceived difficulty (Table 1). Most items fall at the midpoint of the response scale (around 1.5 to 3.0 on the 0 to 4 range). Scenarios involving direct, close communication (e.g., item 5) had the lowest mean response (i.e., least perceived difficulty), while those involving sound localization or listening at a distance (e.g., items 3, 12, 14, and 15) had the highest mean response. All items showed strong, positive item-total correlations, ranging from 0.51 to 0.72. These correlations indicate how aligned each item is with the overall construct of classroom listening difficulty. Overall, the patterns of mean response and item-total correlation values indicate that the VCLASS targets a broad range of classroom listening difficulties, with many everyday school situations perceived as difficult for children with UHL when *not* using hearing aids.

### CTT-Based Item Analyses on the Aided Dataset

In contrast to the unaided condition, response distributions in the aided dataset were shifted toward easier response categories (Table 2), with a large proportion of responses falling into the “very easy” or “a little easy” categories across items. Mean response scores in the aided condition were substantially lower, ranging from 0.40 to 1.12 on the (recoded) 0 to 4 scale, indicating overall improved perceived listening due to hearing aid use. It is intuitive that participants find listening to be easier when using a hearing aid (rather than unaided). Items involving direct, face-to-face communication (e.g., Items 1, 2, 4, and 5) showed the lowest mean response scores (i.e., easiest perceived listening). Items involving complex tasks such as sound localization or selective listening (e.g., Items 12 and 14) retained higher mean response scores, indicating they remained relatively more challenging even with hearing devices (e.g., hearing aids, cochlear implants, bone conduction devices). All items continued to show positive item-total correlations, ranging from 0.43 to 0.67, confirming that items still differentiated between children with higher and lower overall listening difficulty scores while using devices.

Together, these CTT-based mean response and item-total correlations across conditions confirm the VCLASS’s utility. The substantial decrease in mean response from unaided to aided conditions validates the scale’s sensitivity to the intervention of a personal hearing device. The consistently positive item-total correlations in both listening conditions demonstrate the 15 items’ effectiveness in differentiating among children based on their perceived listening difficulty in school settings.

### Phase 2.1: IRT Model Fit Comparisons

All IRT models were fit in R software (R Core Team 2025) using marginal maximum likelihood estimation in the *mirt* package (Chalmers 2012). Missing item responses were imputed using the *mice* package (van Buuren & Groothuis-Oudshoorn 2011). The goal of phase 2.1 was to determine which two-factor IRT model best captured the polytomous response patterns of the VCLASS scale. Identifying the most appropriate two-factor IRT model is an essential step before IRT-based item-level evaluations of the VCLASS. To do so, for each listening condition, we compared two candidate models that are suitable for polytomous data—two-factor GPCM and two-factor GRM—which, as reviewed previously, differ in the kind of constraints imposed on item threshold and discrimination parameters. Because the two-factor GPCM and two-factor GRM are not nested, model comparisons were based on model selection indices: Akaike Information Criterion (AIC; Akaike 1974), Bayesian Information Criterion (BIC), and sample size-adjusted BIC (see Tables 1a and 1b in Supplemental Digital Content, https://links.lww.com/EANDH/C41) that do not require competing models to be nested. These model selection indices are interpreted comparatively for model ranking, rather than against fixed cutoff values; lower AIC, BIC, and sample size-adjusted BIC values indicate a better balance of fit and parsimony. We found that the two-factor GRM provided the best overall balance of fit and parsimony, yielding the lowest AIC (unaided: 40544.51; aided: 23838.94) and BIC (unaided: 40993.58; aided: 24266.06) values, outperforming the two-factor GPCM in both the unaided and aided datasets. As reviewed earlier, the GRM model allows item-specific discrimination parameters and assumes ordered thresholds within an item. Hence, we used the two-factor GRM model for all subsequent item- and test-level IRT analyses.

### Phase 2.2: Parameter Estimates From Best-Fitting IRT Model

Next, we present the final IRT-based estimates using the best-fitting two-factor GRM model for the full 15-item VCLASS for both the unaided and the aided listening conditions. Tables 3 and 4 present item discriminations (*â*^*θ_1_*^ and *â*^*θ_2_*^) and threshold (*β̂_1_*-*β̂_4_*) parameter estimates for all 15 VCLASS items from the two-factor GRM, reported separately for the unaided and aided conditions.

**TABLE 3.** Item discrimination (*â*) and threshold (*β̂*) estimates and standard errors (SEs) for the unaided listening condition

Item	*â^θ_1_^* (SE)	*â*^*θ_2_*^(SE)	*β̂_1_* (SE)	*β̂_2_* (SE)	*β̂_3_* (SE)	*β̂_4_* (SE)
1	−1.553 (0.095)	−0.805 (0.089)	−2.552 (0.134)	−0.791 (0.060)	−0.107 (0.051)	1.494 (0.080)
2	−1.567 (0.100)	−1.034 (0.093)	−2.226 (0.112)	−0.543 (0.054)	0.181 (0.049)	1.428 (0.075)
3	−1.768 (0.103)	0.266 (0.090)	−2.853 (0.158)	−1.504 (0.083)	−0.641 (0.057)	0.581 (0.056)
4	−1.558 (0.114)	−1.581 (0.110)	−2.176 (0.104)	−0.740 (0.053)	0.099 (0.046)	1.445 (0.070)
5	−0.912 (0.199)	−3.907 (0.387)	−1.047 (0.052)	0.127 (0.040)	0.641 (0.044)	1.719 (0.072)
6	−1.310 (0.087)	−0.815 (0.086)	−3.041 (0.177)	−1.047 (0.071)	−0.083 (0.054)	1.499 (0.086)
7	−2.141 (0.115)	−0.259 (0.098)	−2.898 (0.156)	−1.299 (0.069)	−0.370 (0.048)	0.837 (0.055)
8	−2.260 (0.125)	−0.718 (0.106)	−2.695 (0.139)	−1.243 (0.064)	−0.283 (0.046)	1.217 (0.061)
9	−1.479 (0.110)	−1.503 (0.104)	−2.328 (0.115)	−0.569 (0.051)	0.240 (0.047)	1.481 (0.073)
10	−1.487 (0.103)	−1.238 (0.100)	−2.803 (0.153)	−1.157 (0.066)	−0.235 (0.049)	1.311 (0.070)
11	−1.982 (0.137)	−1.896 (0.130)	−1.957 (0.086)	−0.531 (0.046)	0.136 (0.043)	1.400 (0.064)
12	−2.094 (0.116)	0.119 (0.098)	−2.994 (0.163)	−1.657 (0.083)	−0.696 (0.054)	0.550 (0.051)
13	−1.107 (0.085)	−0.915 (0.084)	−2.899 (0.170)	−1.143 (0.077)	−0.029 (0.056)	1.570 (0.092)
14	−2.329 (0.140)	0.469 (0.109)	−2.717 (0.143)	−1.563 (0.077)	−0.820 (0.054)	0.565 (0.050)
15	−2.210 (0.124)	0.000 (NA)	−3.090 (0.172)	−1.338 (0.070)	−0.566 (0.050)	0.447 (0.049)

**TABLE 4.** Item discrimination (*â*) and threshold (*β̂*) estimates and standard errors (SEs) for the aided listening condition

Item	*â*^*θ_1_*^ (SE)	*â*^*θ_2_*^ (SE)	*β̂_1_* (SE)	*β̂_2_* (SE)	*β̂_3_* (SE)	*β̂_4_* (SE)
1	−1.239 (0.212)	2.345 (0.215)	0.003 (0.050)	1.519 (0.082)	2.218 (0.125)	2.992 (0.210)
2	−1.542 (0.275)	3.047 (0.304)	0.234 (0.046)	1.560 (0.079)	2.127 (0.112)	2.639 (0.161)
3	−1.717 (0.134)	0.535 (0.160)	−0.692 (0.069)	1.273 (0.085)	2.302 (0.143)	3.511 (0.263)
4	−1.797 (0.213)	2.124 (0.214)	−0.103 (0.049)	1.352 (0.075)	2.193 (0.120)	3.233 (0.230)
5	−1.405 (0.240)	2.533 (0.245)	0.536 (0.051)	1.716 (0.091)	2.334 (0.135)	3.360 (0.274)
6	−1.704 (0.143)	0.737 (0.161)	−0.223 (0.058)	1.323 (0.087)	2.261 (0.143)	3.402 (0.247)
7	−2.458 (0.175)	0.408 (0.191)	−0.607 (0.059)	1.103 (0.067)	2.024 (0.114)	3.383 (0.261)
8	−2.053 (0.154)	0.806 (0.166)	−0.498 (0.059)	1.265 (0.078)	2.330 (0.138)	3.637 (0.287)
9	−1.519 (0.166)	1.530 (0.152)	−0.077 (0.053)	1.618 (0.095)	2.392 (0.142)	3.560 (0.272)
10	−2.474 (0.180)	0.696 (0.200)	−0.526 (0.056)	1.112 (0.067)	2.028 (0.115)	2.790 (0.174)
11	−1.580 (0.153)	1.260 (0.146)	−0.027 (0.054)	1.580 (0.096)	2.403 (0.146)	3.478 (0.252)
12	−2.539 (0.179)	−0.084 (0.190)	−0.859 (0.064)	0.720 (0.056)	1.741 (0.094)	2.775 (0.182)
13	−1.675 (0.131)	0.607 (0.149)	−0.359 (0.061)	1.381 (0.090)	2.323 (0.145)	3.317 (0.238)
14	−1.988 (0.166)	−0.441 (0.144)	−1.076 (0.077)	1.035 (0.073)	1.808 (0.109)	2.441 (0.157)
15	−1.343 (0.107)	0.000 (NA)	−0.740 (0.082)	1.328 (0.105)	2.227 (0.163)	3.442 (0.274)

#### Item Discrimination Parameter Estimates

Because the model includes two correlated latent factors (factor correlation is *r* = 0.43 in the unaided condition and *r* = 0.54 in the aided condition), each item is characterized by two discrimination parameters (i.e., *â^θ_1_^* and *â^θ_2_^*), representing the effect (slope) of each factor on the item, controlling for the other factor. In the unaided and aided conditions, response patterns were largely captured by a dominant general school-related listening-difficulty factor. Most discrimination estimates exceeded conventional benchmarks for moderate-to-high discrimination (moderate: 0.65 ≤ |*â*| ≤ 1.34; high: 1.35 ≤ |*â*| ≤ 1.69; Baker 2001;De Ayala 2009), indicating that VCLASS items effectively differentiate children across levels of classroom listening difficulty. One item (for the unaided condition) to four items (for the aided condition) loaded more strongly on a specific/minor factor representing school-related close-proximity listening difficulty, as follows. For the unaided condition, item 5 loaded on the specific/minor factor representing school-related close-proximity listening difficulty, whereas for the aided condition, items 1, 2, 4, and 5 loaded on the specific/minor factor representing school-related close-proximity listening difficulty.

#### Item Threshold Parameter Estimates

Because respondents rated each scenario on a five-point polytomous scale, recoded to range from “Very Easy to Hear” to “Very Hard to Hear,” we estimated four threshold parameters per item under the GRM. The thresholds from GRM represent the points along the spectrum of listening difficulty where the probability of selecting a higher response category (e.g., Very Hard to Hear) is larger than that of selecting a lower response category (e.g., A little Hard to Hear). In context, thresholds (*β̂*) reported in Tables 3 and 4 show the transition points that measure how severe a child’s perceived hearing difficulty must be to select higher difficulty categories. Here, we use “lower” and “higher” to describe threshold estimates located toward the lower and upper ends of the observed range. In the unaided condition (Table 3), lower thresholds (e.g., *β̂* values near -3 for Items 6, 12, and 15) indicate that even children with relatively low hearing difficulty were likely to endorse harder response categories for situations involving background noise or sound localization in school environments. Higher thresholds (e.g., *β̂* values around 1.7 for Item 5) indicate that only children with greater listening difficulty tend to report severe hearing challenges in simpler, face-to-face listening situations at school. In the aided condition (Table 4), thresholds were generally shifted toward lower levels of difficulty for items involving direct close-proximity communication (e.g., Items 1, 2, 4, and 5), showing improved perceived listening when hearing devices were used.

To further examine item thresholds, we visually inspected the item characteristic curves (ICC) for each of the 15 items of the VCLASS scale (Figs. 2A, B) for the general listening-difficulty factor under the unaided and aided listening conditions. Each panel presents the probability of selecting each response category from “very easy to hear” to “very hard to hear,” across increasing levels of classroom listening difficulty for the major factor. Items with higher discrimination show steeper ICCs (e.g., Items 7, 8, and 12), whereas flatter curves suggest relatively lower sensitivity and show more gradual transitions between categories (e.g., Item 5). Across items and listening conditions, response curves progress smoothly from left to right (lower to higher categories), which indicates that the GRM thresholds are ordered ascendingly. For instance, the ICC of Item 7 in the unaided condition smoothly progresses from *β̂_1_* = −2.90 < *β̂_2_* = −1.30 < *β̂_3_* = −0.37 < *β̂_4_* = 0.84. This also suggests that the GRM assumptions were met and that as children’s latent listening difficulty increases, they are increasingly likely to choose “very hard to hear.” On the basis of these thresholds and discrimination estimates, these results provide evidence that the VCLASS items are sensitive to detecting a broad range of degrees of classroom listening difficulties among school-age children.

**Fig. 2.**
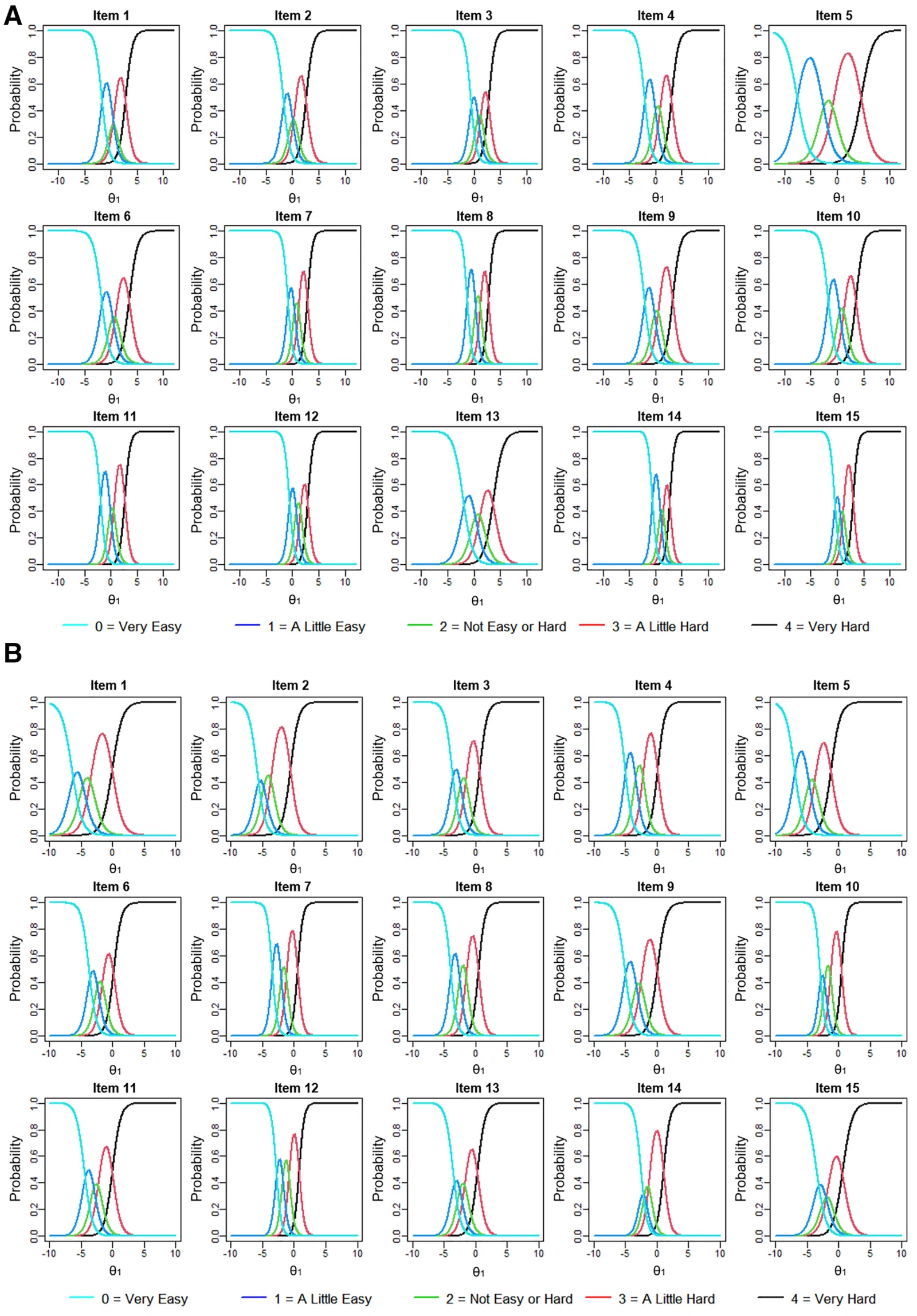
Item characteristic curves (ICCs) for VCLASS Items 1 to 15 in the unaided and aided datasets for the general listening difficulty factor. Panel (A) shows the ICCs of the VCLASS Instrument (Items 1 to 15) for the unaided dataset (for the general listening difficulty factor). Panel (B) shows the ICCs of the VCLASS Instrument (Items 1 to 15) for the aided dataset (for the general listening difficulty factor). The light-blue, dark-blue, green, red, and black curves represent response categories 0 (“Very Easy”), 1 (“A Little Easy”), 2 (“Not Easy or Hard”), 3 (“A Little Hard”), and 4 (“Very Hard”), respectively. For each item, Threshold 1 marks the point (shown by the intersection of the light- and dark-blue curves) at which children are equally likely to endorse category 0 (Very Easy to Hear) versus any higher category (1 to 4). Threshold 2 shows the transition point from endorsing categories 0 or 1 to choosing category 2 or higher. Threshold 3 indicates the point where the likelihood of responding in categories 3 or 4 exceeds that of choosing categories 0 through 2. Threshold 4 shows the point beyond which respondents are more likely to choose the highest category (4, Very Hard to Hear) than any of the lower options (0 to 3). ICC indicates item characteristic curves; VCLASS, Vanderbilt Classroom Listening Assessment Short Survey.

### Phase 2.3 Results: Item Fit Assessment Under Two-Factor GRM

Item-level fit tells us how well each question on the VCLASS survey aligns with the response patterns predicted by the two-factor GRM for each listening condition. In particular, item fit examines whether, across levels of classroom listening difficulty (the latent trait), the observed probabilities of endorsing each response category align with those expected under the model.

We assessed item fit here to identify whether items behave consistently with the latent construct that we aim to measure (classroom listening difficulty). Under the two-factor GRM, for each listening condition, we evaluated item fit with the unweighted mean square fit statistic (*outfit*), the weighted mean square fit statistic (*infit*), and their standardized values (*z*_*outfit*_ and *z*_*infit*_). The infit and outfit statistics provide information about the magnitude of item misfit, with values near 1.0 indicating close agreement between observed and expected responses. Following Adams and Khoo (1996), infit values between 0.75 and 1.33 were considered acceptable, whereas outfit values above 2.00 were considered large enough to potentially distort measurement results (Linacre 2002).

In item-fit analyses (see Table 2 in Supplemental Digital Content, https://links.lww.com/EANDH/C42), all 15 VCLASS items met Adams and Khoo’s (1996) criteria for acceptable item fit (unaided: 0.73 to 1.20; aided: 0.99 to 1.19). In addition, *outfit* values ranged from 0.80 (item 5) to 1.64 (item 11) in the unaided dataset, whereas 0.91 (item 2) to 1.16 (item 10) in the aided dataset. These results demonstrate that all of the items fit the latent constructs for both listening conditions.

### Phase 2.4: Person Fit Assessment Under GRM

For each listening condition, person-fit statistics were used to identify participants whose response patterns deviated from those expected under the two-factor GRM. We evaluated person-fit using the *Zh* index (Drasgow et al. 1985), with |*Zh*| > 1.96 indicating response patterns inconsistent with model expectations at the two-sided *α* = 0.05 level, and |*Zh*| > 2.58 representing the more stringent (*α* = 0.01) criterion. Under the 0.05 criterion, in the unaided dataset, 15.2% were flagged, whereas in the aided dataset, 12.8% were flagged. In both conditions, the majority of flagged cases fell in the moderate *Zh* range (1.96< |*Zh*| <2.58), with few participants showing larger inconsistency (|*Zh*| > 3.00). Overall, these results indicate that most children showed response patterns consistent with the two-factor GRM. The observed misfit levels in both aided and unaided conditions remained within ranges commonly observed in large-scale IRT applications (e.g., see Macia et al. 2023; Weeks et al. 2024; Monaghan et al. 2026).

To determine whether these misfitting response patterns affected our fitted model estimates, for each listening condition, we conducted sensitivity analyses by refitting the two-factor GRM after excluding: (1) respondents flagged with |*Zh*| > 1.96, and (2) a more conservative subset with |*Zh*| > 2.58. In both filtered samples, the GRM parameters in both the unaided and aided analyses remained stable, with the rank order and magnitude of item discrimination (*â*) and threshold (*β̂*_1_ to *β̂*_4_) parameter estimates shifting by no more than 0.3 logits, and all item parameters correlated above 0.98 with the full-sample estimates. Standard errors grew slightly when excluding these overfitting or underfitting cases, which we expected due to the reduced sample size. More importantly, we found that the rank ordering of item discriminations and the shape of the test information curve remained essentially unchanged. These findings indicate that the modest proportion of misfitting patterns does not influence the GRM or our substantive conclusions regarding VCLASS item functioning. Given these minimal differences, we retain and report the full-sample GRM results in the main text to ensure that our findings reflect the entire study population.

### Phase 3: Reliability and Validity

Though internal consistency can be computed within a CTT framework using Cronbach’s alpha—unaided avg. *α* = 0.92 (range 0.91 to 0.92); aided avg. *α* = 0.91 (range 0.90 to 0.91)—in the context of a two-factor model, it is preferable to compute IRT-based marginal reliability because Cronbach’s alpha restrictively assumes unidimensionality and equal true-score variance across items.

#### Marginal Reliability (IRT-Based)

For each listening condition, under the two-factor GRM, we evaluated the reliability of latent factor score estimates using empirical marginal reliability. Reliability was strong for both factors in each listening condition (unaided: 0.88 and 0.89; aided: 0.86 and 0.85 for factors 1 and 2, respectively), indicating good precision in estimating individual differences in classroom listening difficulty. Taken together, results demonstrate that the VCLASS provides reliable measurement across unaided and aided conditions, while appropriately accounting for the multidimensional structure of the scale.

#### Test Information and Measurement Precision

In Fig. 3, we show the dominant general listening-difficulty factor GRM test information curve for Items 1 to 15 across unaided and aided conditions. Each curve summarizes the measurement precision that the VCLASS provides across the latent continuum of classroom listening difficulties. In practice, test information curves are never flat since surveys in real-life scenarios never yield equal information and equal precision across all trait levels. Instead, the information function naturally forms a distribution with an elevated part representing the range of trait values where the VCLASS provides the greatest precision (measurement accuracy). The vast majority of our children had *θ*_1_ scores within these most precise regions. In the unaided condition (where we expect higher listening difficulty), 98% of children had scores between −3.58 and 2.33 (Fig. 3). In the aided condition (where we expect less listening difficulty), 98% of children had scores between −2.42 and 3.76. For the unaided condition, peak information is at 0.34 (with an information value of 13.13). For the aided condition, peak information is at −2.16 (with an information value of 13.98), indicating that the instrument is most precise at measuring higher listening difficulty when participants are unaided.

**Fig. 3.**
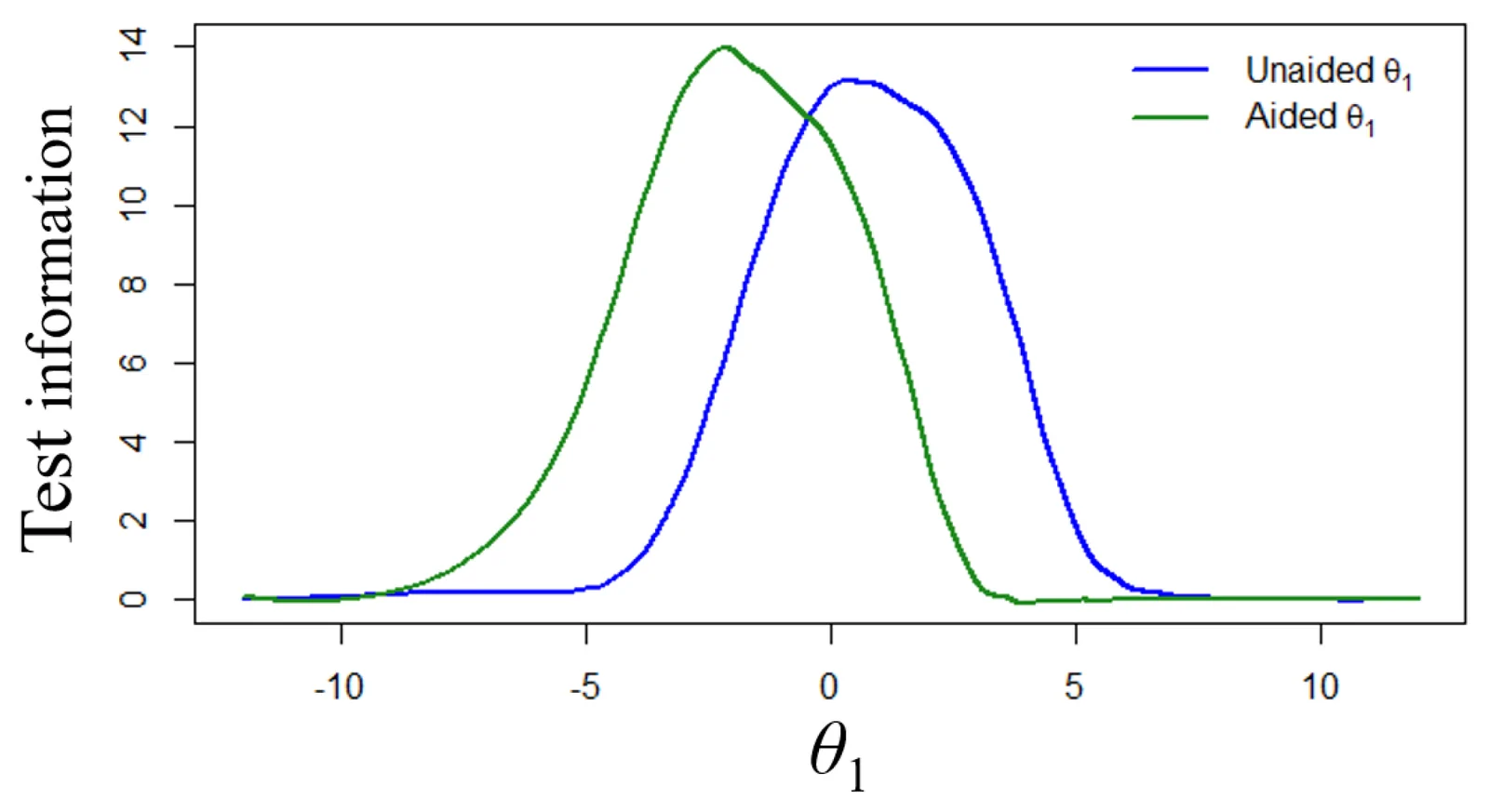
Test Information Curves of the VCLASS Survey (Items 1 to 15) for the unaided and aided condition’s dominant general listening-difficulty factor. VCLASS indicates the Vanderbilt Classroom Listening Assessment Short Survey.

### Phase 4: Results: DIF Analyses on Age

To evaluate whether VCLASS items function equivalently across age for each listening condition, we conducted IRT-based differential item functioning (DIF) analyses using the R *mirt* package (Chalmers 2012) for each of the unaided and the aided listening conditions separately.

### Age-Related DIF

For age-based DIF analysis, we treated age as a continuous variable. For each item, we fitted three nested models:

- Model 1 (baseline): no age term;
- Model 2: adds a main-effect term for *age*, testing whether children’s ratings of the listening situation differ with age;
- Model 3: includes both the *age* main effect and an *Age* × *θ*_1_ and *Age* × *θ*_2_ interaction terms, allowing item discrimination parameters (slopes) to vary by age.

We evaluated DIF using likelihood-ratio tests. Uniform DIF tests the main effects of age-related differences, whereas nonuniform DIF tests whether these differences vary across levels of classroom listening difficulty (that is, the age-by-trait interaction). Total DIF jointly tests both forms (uniform and nonuniform DIF). Comparing model 1 versus model 2 tests for uniform DIF, comparing model 2 versus model 3 tests for nonuniform DIF, and comparing model 1 versus model 3 tests for total DIF. We summarized DIF effect sizes using McFadden’s pseudo‐Δ*R*^2^ change (between each pair of models). According to Zumbo’s (1999) commonly used criteria: negligible (< 0.13), moderate (0.13 to 0.26), or large (> 0.26). Under the more stringent criteria applied by Slocum et al. (2004), DIF effect sizes are classified as: negligible (< 0.035), moderate (0.035 to 0.07), or large (> 0.07). These McFadden’s pseudo-Δ*R*^*2*^ values were used to measure the magnitude of DIF effects, separately for each listening condition (Table 5). Across these 15 items, the magnitude of age-related DIF effects was negligible in both unaided and aided analyses. McFadden’s pseudo-Δ*R*^2^ values for total DIF ranged from 0.000 to 0.011 for the unaided condition, and ranged from 0.000 to 0.022 for the aided condition. These values were also all below Zumbo’s (1999) and Slocum et al.’s (2004) range for moderate DIF, which indicated no age-related bias in the VCLASS item functioning.

**TABLE 5.** McFadden’s pseudo-Δ*R*^2^ effect sizes for age−related DIF analyses

	Unaided DIF Analysis on Age			Aided DIF Analysis on Age		
Item	Uniform DIF Pseudo-Δ*R*^2^	Nonuniform DIF Pseudo-Δ*R*^2^	Total DIF Pseudo-Δ*R*^2^	Uniform DIF Pseudo-Δ*R*^2^	Nonuniform DIF Pseudo-Δ*R*^2^	Total DIF Pseudo-Δ*R*^2^
1	0.0005	0.0033	0.0038	0.0101	0.0074	0.0174
2	0.0001	0.0036	0.0037	0.0053	0.0018	0.0071
3	0.0000	0.0002	0.0002	0.0008	0.0011	0.0019
4	0.0019	0.0070	0.0089	0.0001	0.0008	0.0009
5	0.0012	0.0018	0.0030	0.0065	0.0151	0.0216
6	0.0017	0.0058	0.0074	0.0006	0.0019	0.0025
7	0.0052	0.0061	0.0113	0.0014	0.0009	0.0023
8	0.0007	0.0058	0.0064	0.0000	0.0003	0.0003
9	0.0000	0.0079	0.0079	0.0116	0.0002	0.0119
10	0.0004	0.0019	0.0023	0.0027	0.0056	0.0083
11	0.0011	0.0036	0.0047	0.0023	0.0021	0.0043
12	0.0003	0.0006	0.0009	0.0017	0.0019	0.0036
13	0.0043	0.0063	0.0106	0.0000	0.0013	0.0013
14	0.0015	0.0002	0.0017	0.0012	0.0066	0.0078
15	0.0001	0.0002	0.0002	0.0018	0.0058	0.0076

McFadden’s pseudo-Δ*R*^2^ columns report the corresponding change in pseudo-Δ*R*^2^ for each comparison: comparing model 1 vs. model 2 (uniform DIF), model 2 vs. model 3 (nonuniform DIF), and model 1 vs. model 3 (total DIF).

DIF, differential item functioning.

Overall, DIF analyses do not suggest evidence of uniform or nonuniform age-related DIF items across the VCLASS scale, regardless of whether Zumbo’s (1999) common criteria or Slocum et al.’s (2004) more conservative criteria are used. The negligible pseudo-Δ*R*^2^ values indicate that age is unlikely to affect the interpretation or validity of the VCLASS score. Therefore, there is no empirical justification for excluding any items from VCLASS based on age.

## DISCUSSION

In this study, we applied an IRT-based framework to evaluate psychometric properties of the Vanderbilt Classroom Listening Assessment Short Survey (VCLASS) in 2045 observations from school-age children with UHL who imagined not using (n = 1148) and using (n = 897) personal hearing devices. Unlike the original validation (Picou et al. 2025), we accounted for the ordinal nature of items in all analyses and missing data imputation; we also analyzed the psychometric properties of responses for the unaided and aided responses. Dimensionality analyses supported a two-factor structure for both unaided and aided listening conditions. The best-fitting two-factor GRM showed strong discrimination across all 15 items, indicating that the VCLASS scale can differentiate well between children with varying levels of classroom listening difficulties. In both conditions, the response patterns were explained by a general school-based listening difficulty factor. In addition, one or four items (for unaided and aided conditions, respectively) loaded on a minor/specific factor representing close-proximity listening difficulty. IRT-based reliability indices both showed high internal consistency.

Moreover, we performed DIF analyses, and all McFadden’s pseudo-Δ*R*^2^ values indicated negligible DIF effect sizes across age. This suggests that the VCLASS yields comparable and reliable measurement of classroom listening difficulty across children aged 9 to 17 years in both the unaided and aided conditions, supporting its construct validity and its use in both research and clinical settings.

To our knowledge, this study represents one of the few IRT-based validations of a school-specific listening questionnaire in pediatric audiology. While CTT provides valuable summary indices, the IRT approach offers several unique advantages: sample‐invariant item parameters, interval‐level scoring, test information functions that identify where the scale is most precise, ordered category thresholds, and rigorous detection of DIF. None of these can be gleaned from CTT alone.

### Clinical Implications

Most participants in our sample struggled to hear in some situations (e.g., hearing a teacher from across the room) but not all situations (e.g., face-to-face conversations remained easy). Therefore, we had less data/information for estimating the model-implied scores involving extreme listening disability. This does not imply that the VCLASS scale is invalid for children with extreme listening difficulties. Rather, the model-implied scores for children with extreme listening difficulties would simply have slightly larger standard errors. Clinicians and teachers can use VCLASS as a screening tool to quickly identify students who may benefit from a full audiological assessment or classroom accommodations like preferential seating or remote microphone systems. In addition, the scale can be used to track changes in children’s listening difficulty over time, for instance, before and after the fitting of a personal hearing device or trying a new classroom (intervention) strategy such as preferential seating.

### Limitations and Future Directions

Several limitations should be noted. First, we propose future directions that expand the item coverage targeting extreme levels of hearing difficulties. This would require both recruiting children with more severe listening challenges and developing new, more difficult scenarios to further refine the scale’s precision (measurement accuracy) for children with significant difficulties in classrooms. Second, we investigated DIF across age for both the unaided and aided conditions in this study, but future projects can explore moderation by additional variables such as the degree of hearing loss, laterality of hearing loss (i.e., left vs. right ear), type of device, and levels of self-reported listening fatigue. Moreover, future research could explore how VCLASS performs among children with symmetrical hearing. In particular, future studies can use VCLASS to examine children with symmetrical hearing (both with normal hearing and with bilateral loss). Such studies can provide normative (reference) data for clinical interpretations and help determine whether the patterns and the magnitude of greater difficulty for distance/localization items are similar or different across groups.

## CONCLUSION

Our IRT-based evaluation supports the 15-item VCLASS as a reliable measure of classroom listening difficulty in children with UHL, with and without personal hearing devices. Unique contributions of this study include, for the first time, accommodating the ordinal nature of the data in both analyses and handling missing data, conducting an IRT-based validation separately for aided and unaided conditions, as well as examining DIF by age. Responses were represented by two related factors: a dominant general listening-difficulty factor and a smaller close-proximity listening factor. All 15 items showed good fit, strong discrimination, and high reliability. In addition, age-related DIF was negligible across children aged 9 to 17 years. Test information curves showed that VCLASS was precise across the range of listening difficulty represented by most participants. These results support the use of VCLASS to assess classroom listening difficulty among children with UHL.

## ACKNOWLEDGMENTS

The authors gratefully acknowledge the dedication of our data collection team, including Dr. Anne Marie Tharpe, Dr. Kathleen Healy Lunsford, and Dr. Hilary Davis. The authors also thank Dr. Sun-Joo Cho for her insightful feedback at various stages of the project.

## Supplementary Material

## Footnotes

* For instance, reverse-coding does not change results for parallel analyses, CTT-based item means and item-total correlations, model fit comparisons, GRM item discrimination parameters, slopes of item-characteristics/test-information curves, item/person fit statistics, CTT-based and IRT-based reliability coefficients, and DIF analyses on age. The only changes due to reverse-coding include: reversed ordering of the CTT-based item responses, reversed sign and ordering of GRM threshold estimates, and a mirrored version of item-characteristic/test-information curves.
