# Supplementary material for: An Item Response Theory-Based Validation of the Vanderbilt Classroom Listening Assessment Short Survey for Children With Unilateral Hearing Loss
Source: Ear Hear. 2026 Oct 14;47(6):1674–84. doi: 10.1097/AUD.0000000000001897 (PMC13712651; doi:10.1097/AUD.0000000000001897)
Supplement: Supplementary file 1 [file aud-47-1674-s001.pdf]

Supplemental Digital Content (SDC)
for the manuscript titled: An IRT-based Validation of the Vanderbilt Classroom Listening Assessment Short Survey (VCLASS) for Children with Unilateral Hearing Loss

Supplemental Table 1a. Model fit comparisons between GPCM and GRM for the *unaided* condition

Models	AIC	BIC	saBIC	Log Likelihood
GPCM	40701.51	41150.59	40867.9	-20261.76
**GRM**	**40544.51**	**40993.58**	**40710.89**	**-20183.25**

Supplemental Table 1b. Model fit comparisons between GPCM and GRM for the *aided* listening condition

Models	AIC	BIC	saBIC	Log Likelihood
GPCM	23878.09	24305.21	24022.56	-11850.05
**GRM**	**23838.94**	**24266.06**	**23983.41**	**-11830.47**

Notes on Supplemental Table 1a and 1b. AIC is Akaike's Information Criterion, defined as [(-2) × log-likelihood + 2 × k] (Akaike, 1974), which balances model fit against total estimated parameters, where k is the total number of estimated parameters; BIC is Schwarz's Bayesian Information Criterion, defined as [(-2) × log-likelihood + k × ln(n)] (Schwarz, 1978), imposing a stronger penalty for complexity as sample size (n) grows; saBIC is the sample size adjusted BIC; log-likelihood is the maximized natural log of the maximum likelihood function. **Bold** indicates when the model fit index indicates the better fitting model.
