# Supplementary material for: An Item Response Theory-Based Validation of the Vanderbilt Classroom Listening Assessment Short Survey for Children With Unilateral Hearing Loss
Source: Ear Hear. 2026 Oct 14;47(6):1674–84. doi: 10.1097/AUD.0000000000001897 (PMC13712651; doi:10.1097/AUD.0000000000001897)
Supplement: Supplementary file 2 [file aud-47-1674-s002.pdf]

**Supplemental Digital Content (SDC)**
for the manuscript titled: An IRT-based Validation of the Vanderbilt Classroom Listening Assessment Short Survey (VCLASS) for Children with Unilateral Hearing Loss

**Supplemental Table 1a. Model fit comparisons between GPCM and GRM for the *unaided* condition**

| Models | AIC | BIC | saBIC | Log Likelihood |
|---|---|---|---|---|
| GPCM | 40701.51 | 41150.59 | 40867.9 | -20261.76 |
| **GRM** | **40544.51** | **40993.58** | **40710.89** | **-20183.25** |

**Supplemental Table 1b. Model fit comparisons between GPCM and GRM for the *aided* listening condition**

| Models | AIC | BIC | saBIC | Log Likelihood |
|---|---|---|---|---|
| GPCM | 23878.09 | 24305.21 | 24022.56 | -11850.05 |
| **GRM** | **23838.94** | **24266.06** | **23983.41** | **-11830.47** |

*Notes on Supplemental Table 1a and 1b.* AIC is Akaike's Information Criterion, defined as [(-2) × log-likelihood + 2 × k] (Akaike, 1974), which balances model fit against total estimated parameters, where k is the total number of estimated parameters; BIC is Schwarz's Bayesian Information Criterion, defined as [(-2) × log-likelihood + k × ln(n)] (Schwarz, 1978), imposing a stronger penalty for complexity as sample size (n) grows; saBIC is the sample size adjusted BIC; log-likelihood is the maximized natural log of the maximum likelihood function. **Bold** indicates when the model fit index indicates the better fitting model.
