# Supplementary material for: An Item Response Theory-Based Validation of the Vanderbilt Classroom Listening Assessment Short Survey for Children With Unilateral Hearing Loss
Source: Ear Hear. 2026 Oct 14;47(6):1674–84. doi: 10.1097/AUD.0000000000001897 (PMC13712651; doi:10.1097/AUD.0000000000001897)
Supplement: Supplementary file 3 [file aud-47-1674-s003.pdf]

Supplemental Digital Content (SDC)

for the manuscript titled: An IRT-based Validation of the Vanderbilt Classroom Listening Assessment Short Survey (VCLASS) for Children with Unilateral Hearing Loss

Supplemental Table 2. Item-fit statistics table for the *unaided* & *aided* listening conditions

	Unaided listening condition				***Aided listening condition***			
**Item**	***outfit***	z_{outfit}	***infit***	z_{infit}	***outfit***	z_{outfit}	***infit***	z_{infit}
1	1.283	3.981	1.168	3.475	1.068	.492	1.186	2.439
2	1.423	4.828	1.200	4.048	.913	-.292	1.160	1.909
3	1.051	.705	1.033	.680	1.115	1.351	1.013	.230
4	1.326	3.697	1.172	3.419	1.074	.446	1.125	1.855
5	.800	-2.043	.726	-5.827	.925	-.185	1.101	1.268
6	1.199	3.451	1.177	3.752	.959	-.290	1.050	.781
7	1.307	3.502	1.152	2.985	.994	-.013	1.028	.464
8	1.268	3.233	1.178	3.537	1.001	.046	1.089	1.408
9	1.443	5.449	1.115	2.363	.970	-.179	1.058	.848
10	1.196	2.837	1.161	3.260	1.158	1.064	1.153	2.200
11	1.641	6.276	1.170	3.262	.943	-.354	1.061	.892
12	1.438	4.654	1.080	1.553	1.137	1.450	1.036	.616
13	1.106	1.974	1.130	2.833	.982	-.130	1.012	.199
14	1.016	.203	1.001	.030	1.022	.299	1.101	1.457
15	1.201	1.840	1.040	.802	.954	-.580	.994	-.081
